# Utilization of Google enterprise tools to georeference survey data among hard-to-reach groups: strategic application in international settings

**DOI:** 10.1186/s12942-016-0053-9

**Published:** 2016-07-28

**Authors:** Leo Beletsky, Jaime Arredondo, Dan Werb, Alicia Vera, Daniela Abramovitz, Joseph J. Amon, Kimberly C. Brouwer, Steffanie A. Strathdee, Tommi L. Gaines

**Affiliations:** 1 Northeastern University School of Law and Bouvé College of Health Sciences, 400 Huntington Ave., Boston, MA 02115 USA; 2 Division of Global Public Health, University of California, San Diego, School of Medicine, La Jolla, CA USA; 3 Universidad Xochicalco, School of Medicine, Tijuana, Mexico; 4 Universidad Autonoma de Baja California, Tijuana, Mexico; 5 Health and Human Rights Division, Human Rights Watch, New York, NY USA

**Keywords:** Resource-poor settings, Data collection, Innovation, Vulnerable groups, Liminal spaces, Technology, Tools

## Abstract

**Background:**

As geospatial data have become increasingly integral to health and human rights research, their collection using formal address designations or paper maps has been complicated by numerous factors, including poor cartographic literacy, nomenclature imprecision, and human error. As part of a longitudinal study of people who inject drugs in Tijuana, Mexico, respondents were prompted to georeference specific experiences.

**Results:**

At baseline, only about one third of the 737 participants were native to Tijuana, underscoring prevalence of migration/deportation experience. Areas frequented typically represented locations with no street address (e.g. informal encampments). Through web-based cartographic technology and participatory mapping, this study was able to overcome the use of vernacular names and difficulties mapping liminal spaces in generating georeferenced data points that were subsequently analyzed in other research.

**Conclusion:**

Integrating low-threshold virtual navigation as part of data collection can enhance investigations of mobile populations, informal settlements, and other locations in research into structural production of health at low- or no cost. However, further research into user experience is warranted.

## Background

Dating back to John Snow’s maps of cholera cases in London, the collection of geospatial data is foundational to epidemiology and public health research. In recent decades, however, the advent of geographic information systems (GIS) technology and methodological tools has enabled remarkable advances in the scope, accuracy and power of multilevel analyses of such datasets. This has coincided with the development of novel applications of geospatial analytic methods, including those used to better target programmatic interventions to key populations and enriching our understanding of the relationship between health and our social, cultural, and physical environment, with particular focus on concentrated disadvantage, conflict, and other political and structural determinants of health [1, 2]. Examples of these applications include the use of the built environment to understand obesity, water access and quality research, modeling the diffusion of disease among displaced populations, geolocating adverse law enforcement encounters among drug users to identify service barriers, and a variety of GIS applications to inform supply chains and service delivery [2–11]. More broadly, historic population displacement, mounting globalization, and increased recognition of the interplay of biological, environmental, and structural factors in production of health underscore the significance of georeference data in public health and human rights research.

Despite these opportunities, there remain important challenges in collecting reliable geospatial information, especially in research on mobile and marginalized populations. In survey research, recall of physical address or cross street information may be poor, especially among itinerant, unstably housed, and lower literacy respondents. For instance, in locales of high mobility such as transit hubs, border areas, and refugee havens, survey respondents may be unfamiliar with place names or designations when asked to site particular experiences that occurred within that location. Lack of systematic nomenclature for addresses and street names in certain settings, especially in middle- and lower-income countries hinders precision in georeferenced data collection. Pervasive use of liminal spaces for residential, commercial, and other activity by under-served or criminalized groups (e.g. railroad tracks, canals, informal settlements, etc.) further complicates investigations of the environmental factors shaping their health.

We have experienced many of these challenges first-hand in the context of our research among drug users, sex workers, migrants, and other marginalized and stigmatized groups in the Global South and elsewhere [2, 12–18]. For instance, our research assessing health and human rights domains among people who inject drugs (PWID) along the US–Mexico border typically samples substantial numbers of migrants and deportees who are relatively new to the locales of research [15]. To collect georeferenced data, prior studies relied on paper maps during the interview process to approximate specific locations [7, 15]. As a result, our field staff would expend considerable up-front effort orienting respondents to the map of the locale (as well as—at times—basic cartographic conventions), noting the identified locations using cross-street designation, and later identifying geo-coordinates and transferring these data to the survey database. This laborious process was hampered by limited geographical literacy, as well as open to numerous sources of human error and map imprecision.

The recent advent of free cloud-based mapping tools using satellite and street-view data has proven to be an asset in a variety of research activities, including those in resource-poor settings. In rural sub-Saharan Africa, Google Earth tools have been used to develop a spatial sampling frame to inform subsequent recruitment into a longitudinal survey [9]. In rural Haiti, a combination of Google Earth and GIS software enabled the mapping and random selection of households for water sampling and ethnographic surveys [10]. Among substance users, including PWID, cloud-based mapping tools have been adopted to examine the local areas in which individuals routinely travel and where their daily activities typically occur; to generate geographic coordinates of respondents’ activity spaces, however, these studies relied on a participant’s ability to provide a physical address or nearest street intersection [19, 20].

To our knowledge, no prior published research had employed web mapping technologies as part of the actual data collection process. In an effort to streamline and improve field-based data collection, we developed a novel methodology for the application of Google enterprise tools as part of a larger inquiry into the role of law and law enforcement in shaping infectious disease risk among PWID in Mexico [21]. Thus, the objective of this study is to describe the development and deployment of online mapping technologies in survey research targeting hard-to reach, vulnerable individuals.

## Methods

### Target population

The target population was PWID in Tijuana, recruited as part of a mixed-methods longitudinal study. Study rationale, recruitment, and analytical methods have been detailed elsewhere [5, 22, 23]. Structured interviews identifying the physical spaces in which individuals experienced law enforcement encounters were conducted with those who agreed to participate. The study was approved by the Institutional Review Board at UCSD School of Medicine and Collegio de la Frontera Norte, Tijuana (Project Number 141109).

### Web-based data georeferencing technique

Our quantitative survey instrument assessed sociodemographics, sexual and drug use risk behaviors, migration history, knowledge of criminal laws, and police encounter history. For items related to recent police detention and abuse, we assessed the physical location of the last reported encounter. The instrument was administered in English or Spanish by trained, bilingual interviewers using computer-assisted interview software (QDS™ Systems, NOVA Research, Bethesda, USA).

Our methodological innovation was to integrate Google Enterprise tools including Google Earth and Google Street View into the structured interview protocol. Our laptop workstations running the QDS interview software on a Windows (Microsoft Corporation, Seattle, USA) platform were utilized to assist with georeferencing during data collection. Specifically, when asked for physical location information linked to a particular event (e.g. last instance of physical altercation with a police officer), respondents were able to virtually navigate and pinpoint the location using the integrated Google Street View and Google Earth cloud tools. During this process, the interviewer invited the participant to describe the general area of the encounter, then working with the participant to zoom in and identify the precise location based on narrative description of landmarks and visual anchors. Initially, once the specific location was pinned, interviewers entered the resulting geocoordinates in appropriate data field in the QDS interview database. As the study progressed, we created a software tool that directly transmitted geocoordinate data from the Google Streetview pin to the appropriate field in the QDS database, eliminating the need for human data entry. Paper maps were used as a backup in the 3–5 % of the cases when the Internet connection failed.

### Applications

Our research team conducted multiple geospatial analyses using the georeferenced data collected under the aforementioned protocol. For instance, we triangulated spatial data collected through the innovative technique described above with the Mexican Census to identify concentrated areas of police activity and modeled this relationship to further understand the structural determinants of HIV, hepatitis, and other disease risk among PWID [23]. We also employed geographic weighted regression techniques to determine the spatial association between addiction treatment center locations and the spatial pattern of police interactions with PWID [5]. In addition, we triangulated PWID geospatial data with official crime statistics to identify places of high-risk drug-related activity [24].

## Results and discussion

Our sample covered 737 PWID at baseline, who were 61.9 % male with a median 8 years of education and 50.6 % reporting monthly income equal or less than 2500 pesos (proximately 200 US dollars). Only 37.2 % reported being native to Tijuana, underscoring the prevalence of migrants and deportees in the sample. Our sample reported a median 16 years of injection drug use and 74 % prevalence of incarceration over their lifetime. These characteristics signal high prevalence of mobility, vulnerability and marginalization in the sample, underscoring the instrumental value of the data collection technique described here.

To our knowledge, this study is the first to use cost-free Google enterprise tools to assist participants in identifying geo-coordinates during field data collection. This method provides a low-cost alternative to the modal paper-based georeferencing that poses many challenges, especially among highly mobile and marginalized populations. Unique features of the street navigation and satellite imagery enabled an interactive experience whereby the respondents were able to base their responses on particular geographical features or landmarks without requiring the respondent to be versed in formal nomenclature or the interviewer to have personal familiarity with the full range of possible locales.

The application of these tools proved to be feasible and efficient in terms of minimizing logistical barriers to field collection of georeferenced data, eliminating the cumbersome, labor-intensive, and error-prone utilization of paper maps. Many respondents displayed a high level of engagement and interest in the technology used in this data collection technique. Utilization of the participatory virtual navigation using Google Street view proved almost universally intuitive, even for the many respondents who were not initially familiar with Google Street View or Google Earth tools. This, in contrast to the substantial efforts required to orient many respondents with cartographic conventions during prior studies using paper maps. Further research into user experiences with data collection through participatory victual navigation, including level of coaching necessary, impact of technological literacy, and other elements is warranted.

Through web-based mapping, the research team was able to create a rich dataset of georeferenced encounter points that were subsequently applied in analytical research [5, 23, 24]. Given that these and other, open-sourced mapping tools are freely available on the Internet, this could be a significant methodological innovation, particularly for research in low- and middle-income settings, among itinerant or unstably housed populations, or in liminal spaces. More recent advent of downloadable mapping and navigation applications that can be used off-line (including Google Earth) further extends the utility and promise of this technique, especially in rural and other areas with inadequate or non-existent mobile data or Internet service. At a time of major population mobility and displacement, growth in informal settlements, and increased interest in structural and rights-based determinants of global health, the integration of this technique can serve as a key innovation to streamlining and improving the data collection process.

There are important ethical considerations related to data security and the level of precision of geolocated results. We did not collect identifiable data and no data were actually stored with Google; the mapping software was utilized only to pinpoint the geocoordinates, which were then entered into a separate interview database. Therefore, we did not have privacy concerns about using a publicly-available private enterprise tool for research purposes. However, concerns have been raised that mapping locations of drug use (or sex work, or other clandestine activity by hard-to-reach populations) can provide insight into hotspots of illicit activity [25]. This information can be of interest to law enforcement, who may put pressure on researchers and study participants at risk. Meanwhile, identifiable reports of police abuse can create the risk of police retaliation. Therefore, the protection of both individual as well as community-level data must be considered both when designing and storing datasets containing sensitive georeferenced or other data.

Limitations included human error in recording geocoordinates, difficulty validating recall accuracy, and some challenges familiarizing participants with technological tools. The overall prevalence of missing data for georeferenced items (instances where the respondent reported the police encounter in question, but the geographical information was not provided) was marginally higher than that for other kinds of data in our survey. Across the set of georeferenced items, 17.3–22.8 % respondents answered “do not know” and 1.3–8.8 % refused to answer. Instances where the data was missing for unknown reasons ranged between 2.7 and 3.8 %. Given the sensitivity of the experiences where geo-coordinate information was sought (e.g. instances of physical abuse by police), deploying novel technologies to document precise locations of the encounters may have engendered concerns about possible negative repercussions among some respondents. Whether utilizing geo-referencing technology may impact recall in data collection relating to sensitive or extra-legal behavior or experiences warrants further study. To reduce human error, in subsequent study application of this tool, we have developed a software plug-in that transmits geocoordinate information directly to QDS, minimizing the risk of human data entry error. Open-source tools such as OpenStreetMap are available as alternatives to the use of proprietary Google Enterprise tools described here.

## Conclusion

In this study, using Google Enterprise tools to identify and pinpoint geo-coordinates for survey responses among a sample of PWID proved feasible and efficient. Using this methodology, we were able to operationalize spatial surveillance of the structural determinants of HIV among hard-to-reach populations, making it possible to better target structural public health interventions. Future research should include validation and reliability analyses, cost-effectiveness, and qualitative research drawing on these geospatial data to assess user experiences, respondent confidentiality concerns, and to construct geonarratives.
